# Prediction of pathologic complete response to neoadjuvant systemic therapy in triple negative breast cancer using deep learning on multiparametric MRI

**DOI:** 10.1038/s41598-023-27518-2

**Published:** 2023-01-20

**Authors:** Zijian Zhou, Beatriz E. Adrada, Rosalind P. Candelaria, Nabil A. Elshafeey, Medine Boge, Rania M. Mohamed, Sanaz Pashapoor, Jia Sun, Zhan Xu, Bikash Panthi, Jong Bum Son, Mary S. Guirguis, Miral M. Patel, Gary J. Whitman, Tanya W. Moseley, Marion E. Scoggins, Jason B. White, Jennifer K. Litton, Vicente Valero, Kelly K. Hunt, Debu Tripathy, Wei Yang, Peng Wei, Clinton Yam, Mark D. Pagel, Gaiane M. Rauch, Jingfei Ma

**Affiliations:** 1 Department of Imaging Physics, The University of Texas MD Anderson Cancer Center, 1400 Pressler St., Unit 1472, Houston, TX 77030 USA; 2 Department of Breast Imaging, The University of Texas MD Anderson Cancer Center, Houston, TX USA; 3 Department of Biostatistics, The University of Texas MD Anderson Cancer Center, Houston, TX USA; 4 Department of Breast Medical Oncology, The University of Texas MD Anderson Cancer Center, Houston, TX USA; 5 Department of Breast Surgical Oncology, The University of Texas MD Anderson Cancer Center, Houston, TX USA; 6 Department of Cancer Systems Imaging, The University of Texas MD Anderson Cancer Center, Houston, TX USA; 7 Department of Abdominal Imaging, The University of Texas MD Anderson Cancer Center, 1515 Holcombe Blvd, Unit 1473, Houston, TX 77030 USA

**Keywords:** Cancer, Breast cancer, Cancer imaging, Machine learning, Predictive markers

## Abstract

Triple-negative breast cancer (TNBC) is an aggressive subtype of breast cancer. Neoadjuvant systemic therapy (NAST) followed by surgery are currently standard of care for TNBC with 50-60% of patients achieving pathologic complete response (pCR). We investigated ability of deep learning (DL) on dynamic contrast enhanced (DCE) MRI and diffusion weighted imaging acquired early during NAST to predict TNBC patients’ pCR status in the breast. During the development phase using the images of 130 TNBC patients, the DL model achieved areas under the receiver operating characteristic curves (AUCs) of 0.97 ± 0.04 and 0.82 ± 0.10 for the training and the validation, respectively. The model achieved an AUC of 0.86 ± 0.03 when evaluated in the independent testing group of 32 patients. In an additional prospective blinded testing group of 48 patients, the model achieved an AUC of 0.83 ± 0.02. These results demonstrated that DL based on multiparametric MRI can potentially differentiate TNBC patients with pCR or non-pCR in the breast early during NAST.

## Introduction

Triple-negative breast cancer (TNBC) lacks estrogen receptor, progesterone receptor, and human epidermal growth factor receptor 2 expression, and accounts for about 15% of all breast cancers^1^. TNBC is an aggressive and highly proliferative form of breast cancer and imparts increased risks of distant recurrence and death within 5 years of diagnosis, especially for those patients whose tumor is resistant to standard chemotherapy^2^. Currently, the standard of care for most TNBC patients is surgery preceded by neoadjuvant systemic therapy (NAST), to which about 50–60% of patients have a pathologic complete response (pCR), defined as having no invasive tumor in the breast and in the sampled lymph nodes (the standard definition)^3^. The standard pCR status is an important surrogate marker of breast cancer patients’ clinical outcomes, with an estimated 3-year overall survival rate of 94% for the TNBC patients who have a pCR^4,5^. Another definition of pCR, that no invasive tumor is found in the breast after NAST (breast pCR), is also commonly used. Although the standard pCR is better associated with improved long-term outcomes than breast pCR, the latter is also an important surrogate for long-term outcomes, with an estimated 5-year disease free survival rate of 84% for breast cancer patients^6,7^. However, patients who do not achieve a pCR have significantly worse survival. While there are emerging strategies in the adjuvant setting to further decrease this risk, they do not improve survival estimates as compared with those patients who achieve a pCR to NAST^8^. Therefore, early identification of resistance to NAST that can guide the patients to alternative investigational treatments is a strategy that is currently being extensively explored given the urgent need to maximize pCR, decrease toxicity from ineffective therapies, and improve long-term curability for TNBC patients.

Various MRI acquisitions and analyses have been used to predict the pCR status of patients with breast cancer. These studies reported encouraging results by examining enhancement characteristics or functional tumor volumes from dynamic contrast enhanced (DCE) MRI^9,10^, or by investigating apparent diffusion coefficients (ADCs) from diffusion weighted imaging (DWI)^11,12^. In a study of a cohort of TNBC patients, pCR was weakly associated with the primary tumor shape, peritumoral edema, and signal characteristics from T2-weighted images^13^.

Recently, deep learning (DL) has been increasingly applied to breast cancer diagnosis and treatment response prediction and demonstrated promising results^14,15^. Specifically, a series of studies have been performed to predict response to neoadjuvant chemotherapy in breast cancer patients using DL on PET^16^, MRI^17–20^, or ultrasound^21–23^. However, there are limited reports of using DL for pCR prediction in TNBC patients, and only a small fraction (less than 24%) of the reported studies’ heterogenous cohorts were TNBCs.

In the present study, we aimed to develop a DL network on multiparametric breast MRI to predict the pCR status in the breast specifically for TNBC patients. Using serial DCE MRIs and DWIs that were acquired before NAST and after four cycles of NAST, we developed a three-dimensional (3D) DL network that can extract and fuse the imaging features of multi-timepoint multiparametric MRIs for breast pCR prediction.

## Results

### Patient characteristics

**Figure 1:**
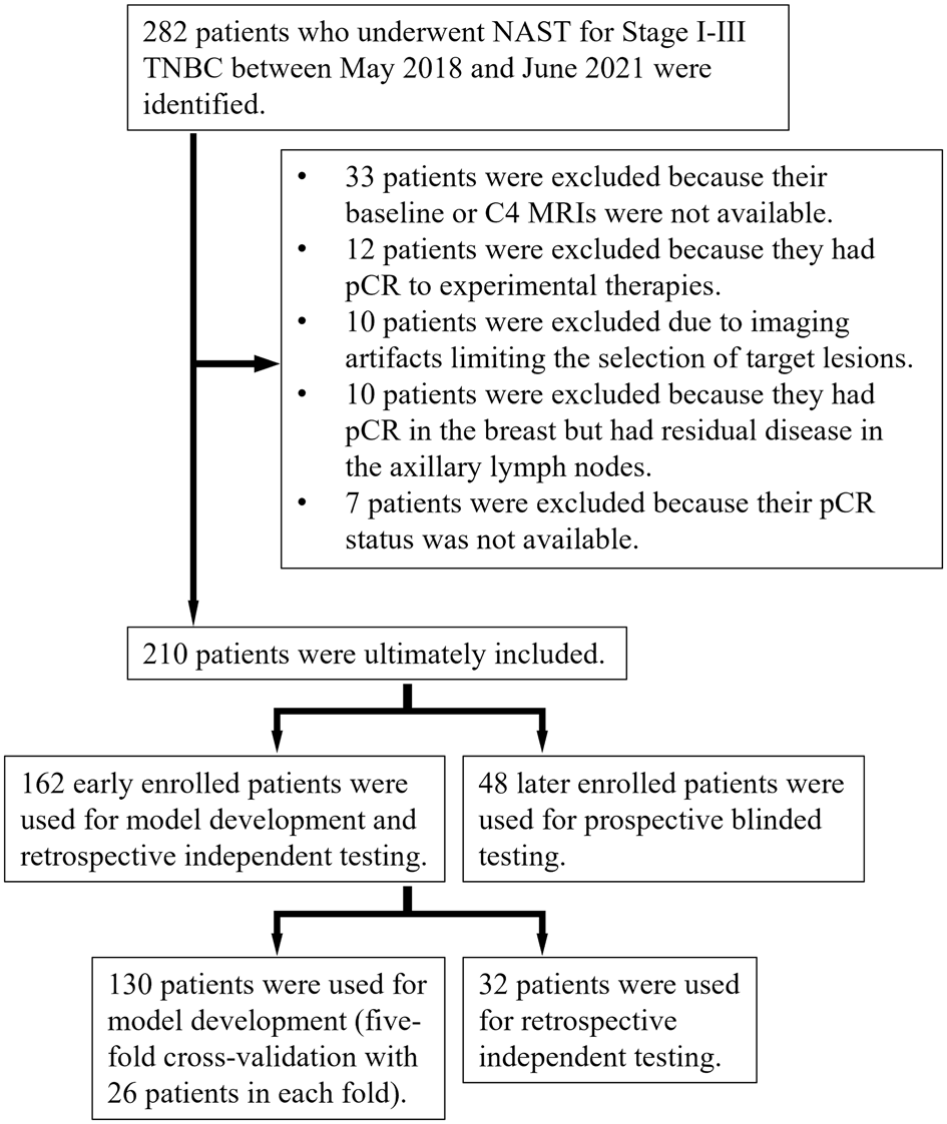
Patient selection. A group of 130 patients was used for model development through fivefold cross-validation. A group of 32 patients was reserved for retrospective independent testing. An extra prospective group of 48 patients was used for blinded testing.

We identified 282 women with stage I-III TNBC who underwent NAST at our institution between May 2018 and June 2021. The NAST regimen consisted of 4 cycles of doxorubicin/cyclophosphamide followed by either paclitaxel or experimental drugs. In total, 210 patients with 210 primary TNBCs met the eligibility criteria and were included in our study (Fig. 1). The mean age (± standard deviation) was 49 ± 11 years and 48% (101/210) had a pCR to NAST. The detailed characteristics of the different patient groups are listed in Table 1.

**Table 1:** Characteristics of the study patients.

	Development group	Testing group (R)	*p*_1_	Testing group (P)	*p*_2_
No. of patients	130	32		48	
Mean age ± SD (y) at diagnosis	48 ± 10	51 ± 13	0.21	50 ± 11	0.31
**Overall clinical stage**					
I	13	4	0.91	10	0.15
II	99	24		33	
III	18	4		5	
**Tumor stage**					
T1	21	5	–	12	–
T2	92	25		32	
T3	14	2		4	
T4	3	0		0	
**Nodal stage**					
N0	88	23	0.50	34	–
N1	29	4		11	
N2	6	3		0	
N3	7	2		3	
Median stromal tumor infiltrating lymphocytes (IQR)	10 (26)	10 (16)	0.48	10 (23)	0.96
Median Ki-67 (IQR)	75 (25)	75 (40)	0.47	75 (40)	0.81
**Histology type**					
Invasive ductal	119	28	0.51	45	–
Metaplastic	8	2		3	
Other	3	2		0	
**pCR status**					
pCR	65	14	0.53	22	0.62
Non-pCR	65	18		26	

Testing group (R): retrospective independent testing group. Testing group (P): prospective blinded testing group. *p*_1_: p-value of the comparison between the development and the retrospective independent testing groups. *p*_2_: p-value of the comparison between the development and the prospective blinded testing groups. The groups’ ages were compared using a t-test; stromal tumor infiltrating lymphocytes and Ki-67 were compared using the nonparametric Mann–Whitney U test; and other non-continuous variables were compared using the chi-squared test.

*SD* standard deviation, *IQR* interquartile range, *pCR* pathologic complete response.

### DL model training and validation

The model was trained and validated on 130 TNBC patients through five-fold cross-validation, with each fold having 26 patients. The 130 patients were randomly selected from the earlier enrolled (before 2021) 162 patients (80%), and the remaining 32 patients (20%) were reserved for independent testing. Among different combinations of the semi-quantitative maps from DCE MRI and DWI, we found that the positive enhancement integral (PEI) map and b800 DWI yielded the best training and validation results. The areas under the receiver operating characteristic curves (AUCs) for the DL model training and validation were 0.97 ± 0.04 and 0.82 ± 0.10, respectively. The training and validation AUCs of using different image combinations are shown in Supplementary Information (SI) Table 1.

### DL model prediction

Using the same image combination of PEI and b800 DWI, we first evaluated the network on the 32 independent testing patients. The averaged AUC for this testing group was 0.86 ± 0.03. Using the probability thresholds established by the development group, the model had a prediction accuracy of 77 ± 4%, a sensitivity of 77 ± 10%, a specificity of 77 ± 10%, a positive predictive value (PPV) of 73 ± 8%, and a negative predictive value (NPV) of 82 ± 6%.

**Figure 2:**
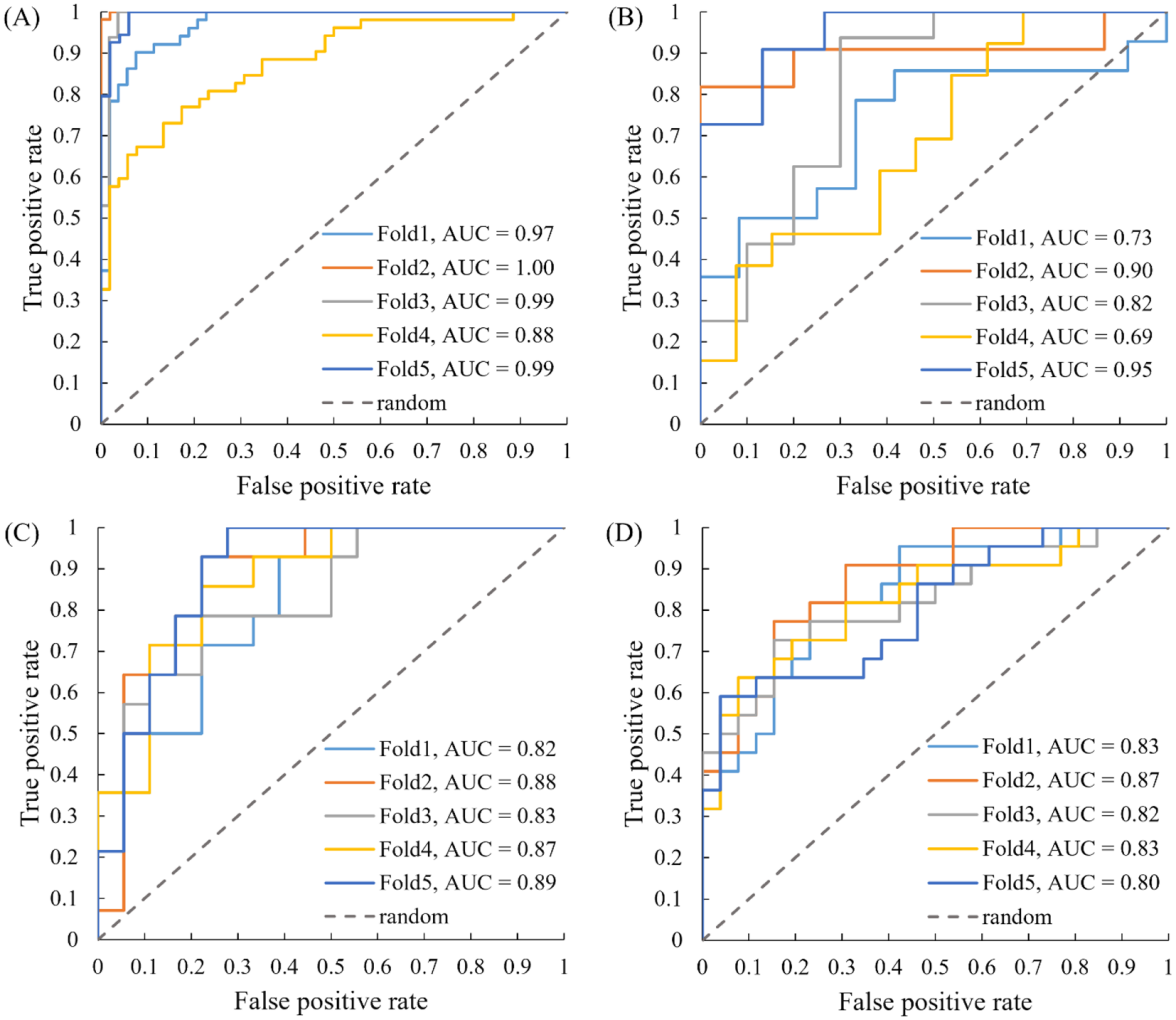
The receiver operating characteristic (ROC) plots and corresponding areas under the curves (AUCs) of each fold for the training (**A**), validation (**B**), retrospective independent testing (**C**), and prospective blinded testing (**D**) groups. The averaged AUCs for each group were 0.97, 0.82, 0.86, and 0.83, respectively. The training and validation groups were used to develop the deep learning network and determine the optimal operating thresholds.

To further validate the generalization and applicability of the DL model, we evaluated the DL network on a prospectively acquired blinded testing group. The prospective testing group consisted of 48 TNBC patients who were enrolled after 2020 in our clinical trial, and the breast pCR status was blinded to the DL network developer. The averaged AUC for the prospective testing group was 0.83 ± 0.02. Using the same probability thresholds as in the initial testing group, the model had a prediction accuracy of 74 ± 3%, a sensitivity of 60 ± 6%, a specificity of 86 ± 4%, a PPV of 79 ± 5%, and an NPV of 72 ± 3%. The detailed results of the model training, validation, and prediction performance are listed in Table 2, and the receiver operating characteristic curves (ROCs) are plotted in Fig. 2.

**Table 2:** Deep learning model prediction of breast pCR in the training, validation, retrospective independent testing, and prospective blinded testing groups.

Groups	AUC	Accuracy	Sensitivity	Specificity	PPV	NPV
Training	0.97 ± 0.04	94 ± 7%	91 ± 13%	95 ± 3%	95 ± 3%	93 ± 10%
Validation	0.82 ± 0.10	88 ± 10%	77 ± 20%	83 ± 13%	84 ± 9%	80 ± 12%
Testing (R)	0.86 ± 0.03	77 ± 4%	77 ± 10%	77 ± 10%	73 ± 8%	82 ± 6%
Testing (P)	0.83 ± 0.02	74 ± 3%	60 ± 6%	86 ± 4%	79 ± 5%	72 ± 3%

*pCR* pathological complete response, *Testing (R)* retrospective independent testing, *Testing (P)* prospective blinded testing, *AUC* area under the curve, *PPV* positive predictive value, *NPV* negative predictive value.

### Tumor volume change between pCR/non-pCR patients

To give more clinical perspectives, we calculated the tumor volume change and volume percentage change according to MRIs between the baseline and after 4 cycles of NAST (C4). As shown in Table 3, the tumor volume change between pCR and non-pCR patients was not significant for all the development, retrospective testing, and prospective testing groups (p = 0.11, 0.40, 0.93, respectively). However, the percentage volume change compared to baseline MRIs showed significant difference for all three groups (p < 0.01) between the pCR and non-pCR patients.

**Table 3:** Tumor volume change between the baseline and C4 MRI for the development, retrospective independent testing, and prospective blinded testing groups.

Groups	Tumor volume change (cm^3^)			Percentage change (%)		
	pCR patients	Non-pCR patients	*p*	pCR patients	Non-pCR patients	*p*
Development	− 11 ± 13	− 16 ± 21	0.11	− 89 ± 10	− 69 ± 28	< 0.01
Testing (R)	− 12 ± 8	− 24 ± 56	0.40	− 92 ± 14	− 70 ± 26	< 0.01
Testing (P)	− 13 ± 16	− 13 ± 18	0.93	− 89 ± 26	− 61 ± 30	< 0.01

*pCR* pathological complete response, *Development* patients for model training and validation, *Testing (R)* retrospective independent testing, *Testing (P)* prospective blinded testing.

## Discussion

Our work showed that based on serial multiparametric MRIs, DL can predict the breast pCR status of TNBC patients receiving NAST early with good performance. Our DL model is different from and has several advantages over the models reported in other studies. First, rather than using one MRI acquired at a single timepoint, we incorporated DCE MRI and DWI acquired at baseline and at C4 for breast pCR prediction. The MRIs acquired at different timepoints can provide better characterization of longitudinal changes of the primary tumors in the breast and their response to treatment. Moreover, DWI is sensitive to tissue cellularity and can provide complementary information to DCE MRI in breast pCR prediction as reported in a previous study based on the I-SPY 2 trial^12^. Second, our model requires as inputs only tumor ROIs with bounding boxes instead of the exact tumor segmentation, which renders it easier for clinical implementation because tumor segmentation is often performed manually by trained experts and can be subjective and labor-intensive. Third, instead of using a single slice or multiple slices, our DL model uses an ROI that encompasses the entire tumor volume in the breast and performs 3D convolution for imaging feature extraction. Using 3D tumor volumes is more advantageous because selecting image slices through the tumor can be subjective. More importantly, a 3D volume contains more complete information on the intra-tumoral heterogeneity and spatial features that are extracted by a DL network.

We investigated different combinations of DCE MRI semiquantitative maps and DWI as inputs to the DL model and found that the PEI map and b800 DWI yielded the best prediction performance. We found that replacing PEI with MSI (maximum slope of increase) or SER (signal enhancement ratio) resulted in noticeably lower performance in either the training, validation, or retrospective testing group (SI Table 1). The reasons behind these findings are unclear and may require further studies for elucidation. Nonetheless, the semiparametric PEI map is a reliable metric for characterizing the fast uptake of the contrast agents by a tumor that reflects the tumor angiogenesis and vascularity by DCE MRI^24,25^. Additionally, replacing b800 DWI with lower b-value DWI or the apparent diffusion coefficient **(**ADC) maps also yielded decreased performance, which may be due to the low diffusion contrast of the low-b value DWI, or the enhanced noise of the ADC maps.

In addition to the retrospective testing group, our DL model was also evaluated on the prospective blinded testing group. The total number of patients of the two testing groups was 80, nearly 40% of our total TNBC patients. Further, the performance of predicting breast pCR for the prospective testing group was similar with that for the retrospective testing group (AUCs 0.83 vs. 0.86, p = 0.16; prediction accuracies 74% vs. 77%, p = 0.33), indicating that our model was stable and can potentially be applied to future patients.

The significant volume percentage change may also help distinguish between pCR and non-pCR patients. This was consistent with our image preprocessing and multi-timepoint inputs strategy, suggesting that our developed DL model may have captured the percentage volume change between the longitudinal MRI scans. However, given the large standard deviation of the percentage volume change, this single metric may have low precision to predict the pCR status.

Besides the conventional analyses, several studies have reported promising results of using DL for breast cancer treatment response prediction. However, most of these reports were for heterogenous breast cancer subtypes and less than 24% of the included patients had TNBC, which may have substantially different imaging features from other breast cancers and the pCR prediction for TNBC can be more challenging^26^. Based on 42 breast cancer patients, El Adoui et al. developed a DL model for pCR prediction by using the pre- and post-treatment DCE images as inputs and achieved an AUC of 0.91 on 14 testing patients^17^. Ha et al. developed a DL model using the pre-treatment postcontrast T1-weighted images of 113 breast cancer patients. Evaluated on 28 patients, the model had an overall prediction accuracy of 88% in classifying the patients into complete, partial, and no response groups^18^. Qu et al. also incorporated pre- and post-treatment DCE images of 244 breast cancer patients for DL model development and achieved an AUC of 0.97 on 58 testing patients^20^. However, all these studies used 2D image slices for model training. Although some studies achieved high AUCs, the results may not be generalizable because of the heterogenous patient population and the small number of training or testing patients. Furthermore, for the reported studies using DCE MRI, only images from a single or a few temporal phases of DCE MRI were used. Joo et al. developed a DL model based on the pre-treatment T1-weighted images, T2-weighted images, and clinical information of 429 breast cancer patients^19^. Their model performed 3D convolution on entire bilateral image volumes including the chest wall and achieved an AUC of 0.89 on 107 testing patients. However, the percentage of TNBC patients in their study population was not specified.

Our study has several limitations. First, the training and testing of the current DL model excluded the patients who had breast pCR but had residual disease in the axillary lymph nodes (ALNs) (Fig. 1). These patients accounted for only a small fraction of our study population (10 out of 220), but they are generally included in the standard clinical definitions of non-pCR. When these patients were included in our model development and testing, the performance of the model was not much affected, but it became noticeably lower in the prospective testing group (SI Table 2). These findings could be due to the difficulty in predicting the axillary nodal status using the images of only the primary tumors, and/or to the small number of the patients that were available. Sun et al. showed that DL on ultrasound images including the peritumoral regions can help predict ALN metastasis for breast cancer^27^. Similarly, Zheng et al. showed that DL on the ultrasound images and the shear wave elastography images of breast cancer can also predict ALN metastasis^28^. Therefore, the performance of our model in predicting the status of ALN may be improved with the inclusion of peritumoral regions on MRI or ultrasound images.

Second, our model used imaging data only. Several recent studies have shown that other information such as patient clinical (including pathological/immunohistochemical) or genomic data may be useful for pCR prediction in breast and rectal cancers^19,29–31^. Therefore, our model could be further improved by including patient clinical and/or genomic data. Third, the current sample size was small, and we had to rely on multi-fold cross-validation to thoroughly demonstrate the testing performance. Although multi-fold cross-validation can also be used for prospective prediction by majority voting^32^, a single classifier trained by a large dataset may be preferred. In the future, more data will be collected, which will also help to improve the model performance. Finally, our model currently requires the use of baseline and C4 images. A preliminary study showed that using baseline images only the testing prediction AUC dropped markedly to 0.65. Therefore, longitudinal images should be necessary for better prediction. We plan to investigate the use of earlier MR images, such as after 2 cycles of NAST, to develop a DL model for breast pCR status prediction. This would allow earlier interventions or changes to the treatment strategy and provide an increased benefit for TNBC patients.

In conclusion, we developed a 3D DL network that uses multiparametric MRIs acquired at baseline and after four cycles of doxorubicin/cyclophosphamide treatment to predict breast pCR to NAST in TNBC patients. The DL network achieved high and consistent prediction performance and can be implemented without requiring exact tumor segmentation. It has the potential to help guide the management of TNBC patients and triage those with NAST resistance for investigational treatment alternatives and precision medicine strategies.

## Methods

This study was approved by the Institutional Review Board of The University of Texas MD Anderson Cancer Center and is part of an ongoing clinical trial of patients with stage I-III TNBC who were prospectively monitored for response to NAST. All methods were carried out in accordance with the Declaration of Helsinki. Written informed consent was obtained from each participant.

### Treatment regimens and MRI acquisitions

NAST included 4 cycles of dose-dense doxorubicin (Adriamycin)/cyclophosphamide (Cytoxan) (A/C) treatment followed by 12 cycles of paclitaxel treatment. Patients who did not respond well to A/C treatment were switched to experimental drugs that included Atezolizumab, Panitumumab, Enzalutamide, and Everolimus. After completing NAST, patients underwent surgical resection. The assessment of the resected specimen by pathology for any residual disease was used to establish the patient’s pCR status. Multiparametric MRIs acquired at two timepoints: (1) pre-treatment (baseline), (2) after 4 cycles of A/C treatment (C4), were included in our study. The MRIs of both timepoints were used for DL network development.

### Histopathology review

Core needle biopsy specimens of the primary tumors before treatment were obtained for immunohistochemical assessment and assessment of histological type. Estrogen receptor (ER), progesterone receptor (PR), human epidermal growth factor receptor 2 (HER2), and Ki-67 results were reported as the percentage of cells with positive nuclear staining. TNBC was defined as a tumor in which < 10% of invasive tumor cells had positive nuclear staining for ER and < 10% of cells had positive nuclear staining for PR^33^. According to the American Society of Clinical Oncology/College of American Pathologists guidelines, HER2 negativity was defined as the immunohistochemistry assay or the in situ hybridization assay of the cancer specimen was negative^34^.

### Study participants

Patients were excluded from the study if their baseline or C4 MRIs were not available; their MRIs had artifacts; their pCR status was not available; they had no residual disease in the breast but had residual disease in the sampled ALNs; or if they had a pCR to an experimental therapy. Note that the patients who did not achieve a pCR to experimental therapies were included in our study, as these patients did not respond to A/C regimen and were not expected to respond to the paclitaxel in the first place and thus could be safely assumed to have non-pCR if they were treated with paclitaxel. We used imaging data from the early enrolled 162 patients (before the end of 2020) for DL network development and retrospective independent testing. The imaging data from an extra set of 48 patients who were later enrolled (after the end of 2020) were used for prospective blinded testing of the DL model.

### MRI scan parameters

All patients were scanned on a 3T GE Discovery 750w scanner (GE Healthcare, Waukesha, WI) using an 8-channel bilateral phased array breast coil. DCE MRI was acquired with a 3D T1-weighted DISCO sequence with a dual-echo bipolar readout for fat–water separation in conjunction with an intravenous bolus injection of the contrast agent (Gadovist, Bayer HealthCare, Whippany, NJ; 0.1 mL/kg at ~ 2 mL/s followed by saline flush). Typical scan parameters for the DISCO acquisition were as follows: matrix size 320 × 320; field of view 30 × 30 cm^2^; slice thickness 3.2 mm; slice spacing − 1.6 mm; flip angle 12°; echo times 1.1/2.3 ms; repetition time 6 ms. Temporal resolution of the DCE MRI was approximately 12 s. The total number of slices was about 120, and the total number of temporal phases ranged from 32 to 64. DWI was acquired prior to DCE MRI with a reduced field-of-view single-shot echo planar imaging sequence (FOCUS, GE Healthcare). Typical scan parameters for DWI were as follows: matrix size 80 × 80; field of view 16 × 16 cm^2^; slice thickness 4 mm; slice gap 0 mm; flip angle 90°; echo time 70 ms; repetition time 4 s. Two b-values used were 100 and 800 s/mm^2^, and the total number of slices was 16. Although the DCE and DWI had different in-plane resolutions, the reconstructed images had similar image resolution (0.59 and 0.63 mm/pixel, respectively) by zero-padded reconstruction and therefore had similar ROI size.

### Image curation

In our study, we primarily focused on the wash-in periods of the DCE images because the signal plateau was relatively easy to define. After selecting the early enhancement frames characterizing the wash-in phase, three semi-quantitative maps: positive enhancement integral (PEI), maximum slope of increase (MSI), and signal enhancement ratio (SER) were generated on an AW Server (v3.2, GE Healthcare, Milwaukee, WI). PEI was calculated by summing up the signal intensities (SI) of each frame (PEI = ∑SI_t_)^35^, MSI was obtained by selecting the maximum rate of SI increase between frames (MSI = max(SI_t_ − SI_t−1_)), and SER was calculated as the ratio of differences involving the plateau, last, and precontrast frames (SER = (SI_plateau_ − SI_pre_)/(SI_last_ − SI_pre_))^36^.

**Figure 3:**
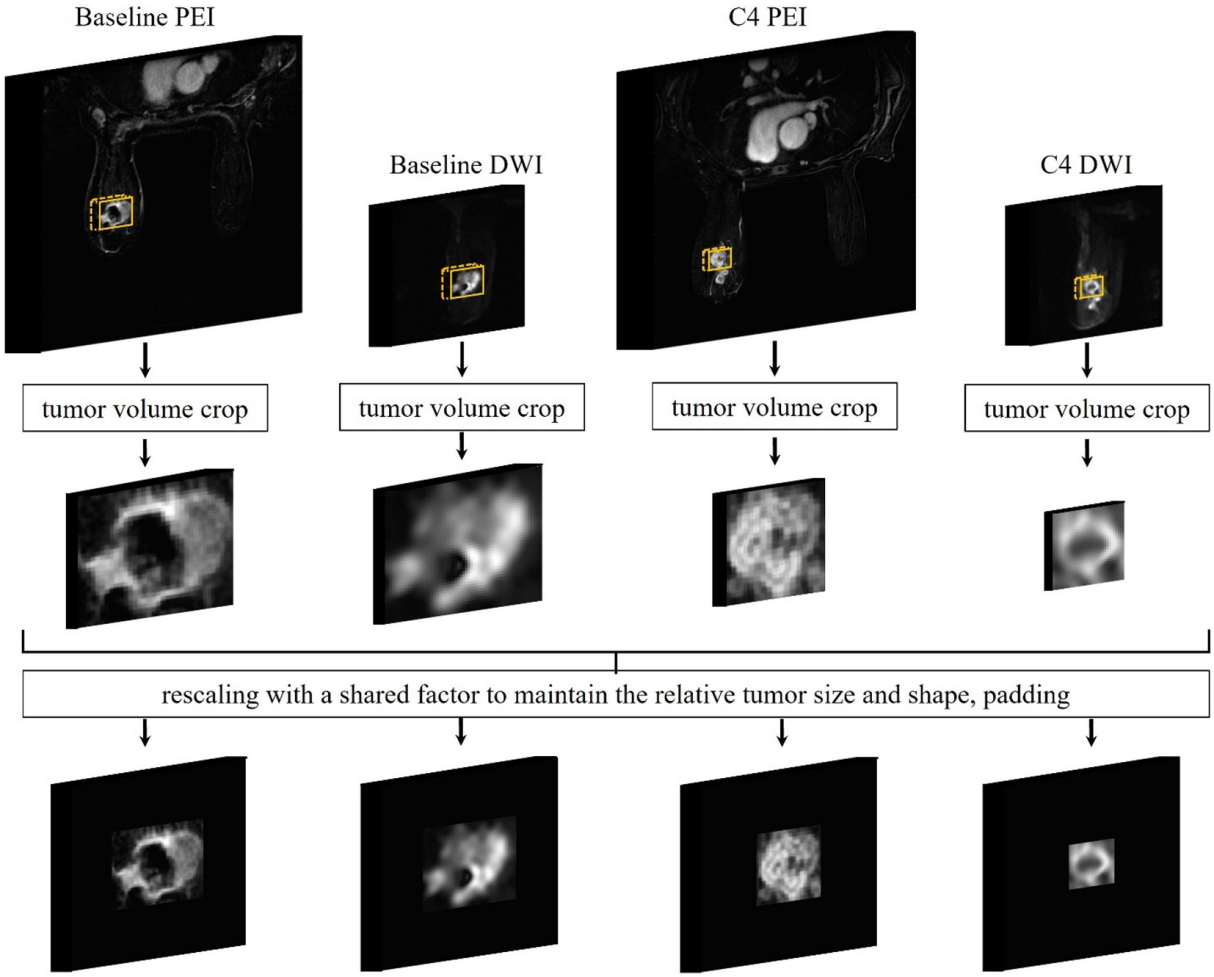
The image curation process. The tumors were cropped by placing tight bounding boxes based on the segmentations. The baseline tumor crops were first rescaled to the median size, then we used the same rescaling factor to resize the C4 tumor crops. Finally, all rescaled crops were zero-padded to the same sizes of 80 × 80 × 24 for the PEI, and 80 × 80 × 12 for the DWI. Such preprocessing approach permits maintaining the relative tumor shape across the patients, maintaining the relative tumor size across two scans, and avoiding large amounts of zero-padding, which can be more efficient for model training. The signal intensities were then normalized between 0 and 1.

All MRIs were first segmented by 3 experienced radiologists (M.B., R.M.M., S.P.) with consensus. The tumor volumes were then cropped based on the segmentation by placing 3D tight bounding boxes surrounding the tumors. Some peri-tumoral tissues might be included however they had low signal intensity. In our study, the median tumor size of the baseline scan was 2.9 cm (range 1.2–11 cm). To compensate for the large size and shape variation, we performed “size-normalization” before inputting the crops to the DL model. Compared to simple zero-padding or resizing the crops to the same input size, our normalization method maintained the relative tumor shape across the patients and maintained the relative tumor size across their two scans. It also avoided large amounts of zero-padding, which can be more efficient for DL model training (Fig. 3). Finally, the signal intensities of all image crops were normalized between 0 and 1.

### Data partition

To mitigate the limitation of the relatively small sample size and reduce possible training bias, we used five-fold cross-validation in our DL model development. The first 162 patients were randomly partitioned into development and retrospective testing groups with the ratio of 130:32 (80%:20%). The imaging data of the development group was used for model training and validation, and the data of the retrospective testing group was reserved for independent model evaluation. For the development group, the 130 patients were randomly split into five folds with 26 patients per fold. Iteratively, four of the five folds were merged for model training and the remaining fold was used for validation to prevent model overfitting. In total, the model was trained five times and tested in the retrospective testing group.

Besides the retrospective testing group, we evaluated the performance of the DL model in another group of 48 patients who were subsequently enrolled in the clinical trial (after the end of 2020). The images of the prospective group were not exposed to the DL model during its development. Further, the pCR status of these patients was blinded to the DL model developers. The performance evaluation was carried out and verified by independent investigators.

### Construction of DL networks

**Figure 4:**
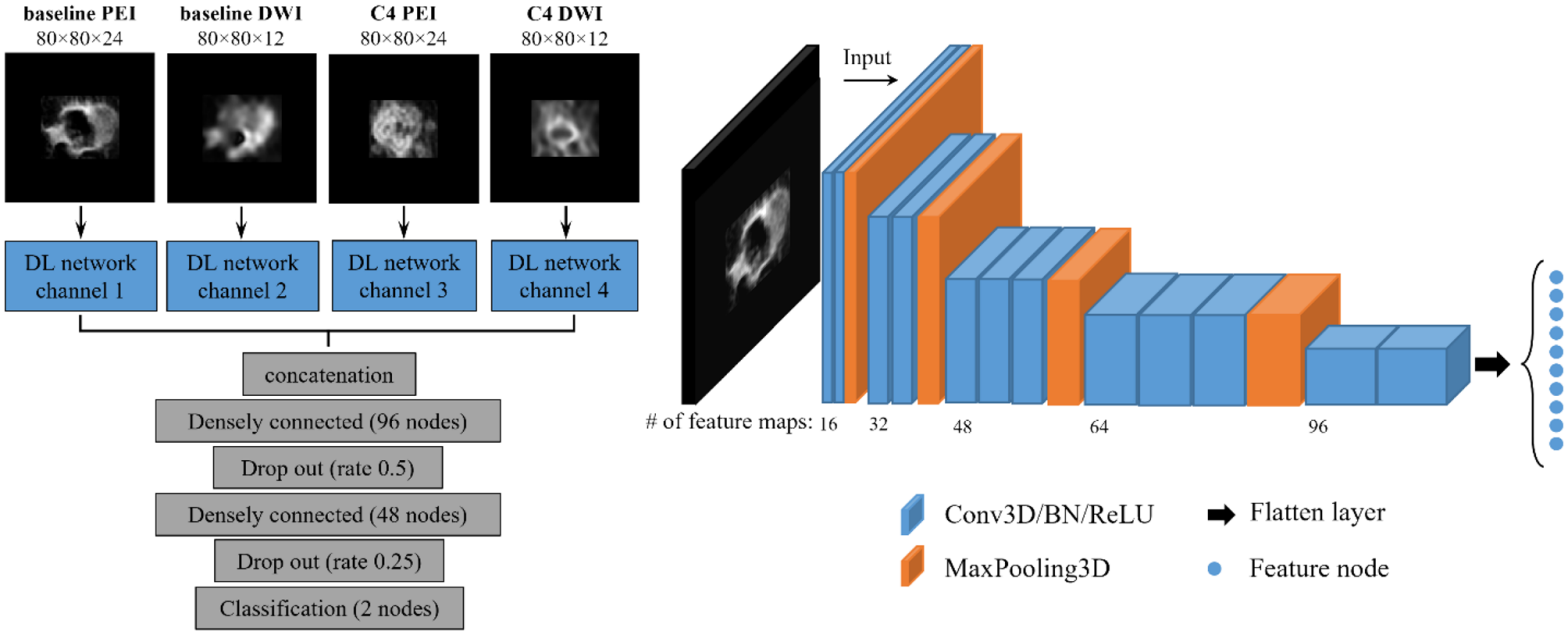
The deep learning network. The framework of the feature extraction module, shown at left, consisted of four input channels corresponding to baseline positive enhancement integral (PEI), baseline diffusion-weighted imaging (DWI), PEI acquired after 4 cycles of doxorubicin/cyclophosphamide treatment (C4 PEI), and DWI acquired after 4 cycles of doxorubicin/cyclophosphamide treatment (C4 DWI). The detailed structure of the feature extraction module of one channel is shown at right. The extracted features of the four channels were concatenated and used for breast pathologic complete response prediction. Two dropout layers were added after the densely connected layers to prevent overfitting of the model. Conv3D: 3D convolutional layer, BN: batch normalization, ReLU: rectified linear unit, MaxPooling3D: 3D max pooling layer.

The DL network was designed to use multiple images from both the baseline and C4 acquisitions as inputs (Fig. 4). For each channel, the feature extraction module was constructed using stacked convolutional layers, batch normalization layers, and MaxPooling layers. We used 3D convolutions to extract features from entire tumor volumes. Following convolution and batch normalization, rectified linear unit (ReLU) was used as the activation function. For the MaxPooling layers, the pooling window size was 2 × 2 × 2 or 2 × 2 × 1 according to the feature map sizes at different layer depths of each input channel. The final size of each extracted feature map was 5 × 5 × 1.

The feature maps of each channel were then flattened and concatenated before being forwarded to 3 densely connected layers for pCR prediction. The first 2 dense layers were followed by dropout layers with dropout ratios of 0.5 and 0.25, respectively, to prevent overfitting and increase the network’s generalization. The final dense layer was the classification layer. Using Softmax activation, the classification layer assigns probabilities of each patient having a breast pCR.

### Network training configuration

Categorical cross-entropy loss was used as the loss function to train the network. The loss was optimized using an Adam optimizer with an initial learning rate of 1 × 10^–5^. The learning rate was decreased by 0.1 if the validation loss did not improve in 3 consecutive training epochs. The training process was terminated if the validation loss did not improve in 7 consecutive training epochs. The model with the minimal validation loss was saved for testing.

To prevent overfitting and to improve the generalization of the model, besides dropout layers, we used random affine transformations to augment the input images 12 times during training. The affine transformations included random flip in the horizontal direction, translation within 5 pixels, and scaling with a range of 0.8–1.2. The same transformation was used for all input channels during data augmentation.

### Network evaluation and statistical analysis

The models trained by each iteration of cross-validation were independently tested using the testing groups. ROC curves and AUCs were determined for each model. For both the retrospective and prospective testing groups, accuracy, sensitivity, specificity, positive predictive value (PPV), and negative predictive value (NPV) were calculated based on the probability thresholds established by the development group.

### DL model training and evaluation based on the standard pCR definition

Besides the analysis based on pCR in the breast, we performed a preliminary study using the standard pCR definition by including the 10 patients who had no residual disease in the breast but had residual disease in the ALNs. These patients were considered non-pCR under the standard pCR definition. To evaluate the model’s prediction performance after including these patients, we retrained and re-evaluated the model on both the retrospective and the prospective testing groups. The results were shown in the supplementary information.

## Supplementary Information

Supplementary Information.

## Data Availability

The data are not publicly available as they contain information that could compromise research participant privacy or consent. The DL model code developed for this study can be available upon request from the corresponding authors Dr. Gaiane M. Rauch and Dr. Jingfei Ma.
