# Supplementary material for: Prediction of pathologic complete response to neoadjuvant systemic therapy in triple negative breast cancer using deep learning on multiparametric MRI
Source: Sci Rep. 2023 Jan 20;13:1171. doi: 10.1038/s41598-023-27518-2 (PMC9859781; doi:10.1038/s41598-023-27518-2)
Supplement: Supplementary file 1 — Supplementary Information. [file 41598_2023_27518_MOESM1_ESM.docx]

Supplementary Information for

**Prediction of pathologic complete response to neoadjuvant systemic therapy in triple negative breast cancer using deep learning on multiparametric MRI**

Zijian Zhou^1^, Beatriz E. Adrada^2^, Rosalind P. Candelaria^2^, Nabil A. Elshafeey^2^, Medine Boge^2^, Rania M. Mohamed^2^, Sanaz Pashapoor^2^, Jia Sun^3^, Zhan Xu^1^, Bikash Panthi^1^, Jong Bum Son^1^, Mary S. Guirguis^2^, Miral M. Patel^2^, Gary J. Whitman^2^, Tanya W. Moseley^2,5^, Marion E. Scoggins^2^, Jason B. White^4^, Jennifer K. Litton^4^, Vicente Valero^4^, Kelly K. Hunt^5^, Debu Tripathy^4^, Wei Yang^2^, Peng Wei^3^, Clinton Yam^4^, Mark D. Pagel^6^, Gaiane M. Rauch^2,7*^, Jingfei Ma^1*^

Departments of ^1^Imaging Physics, ^2^Breast Imaging, ^3^Biostatistics, ^4^Breast Medical Oncology, ^5^Breast Surgical Oncology, ^6^Cancer Systems Imaging, ^7^Abdominal Imaging, The University of Texas MD Anderson Cancer Center, Houston, TX

*Correspondence:

Gaiane M. Rauch, Department of Breast Imaging and Abdominal Imaging, The University of Texas MD Anderson Cancer Center, 1515 Holcombe Blvd, Unit 1473, Houston, TX, 77030. Email: [gmrauch@mdanderson.org](mailto:gmrauch@mdanderson.org).

Jingfei Ma, Department of Imaging Physics, The University of Texas MD Anderson Cancer Center, 1400 Pressler St., Unit 1472, Houston, TX, 77030. Email: [jma@mdanderson.org](mailto:jma@mdanderson.org).

*Abbreviations:* deep learning (DL), triple negative breast cancer (TNBC), neoadjuvant systemic therapy (NAST), pathologic complete response (pCR), positive enhancement integral (PEI), maximum slope of increase (MSI), signal enhancement ratio (SER), diffusion weighted imaging (DWI), area under the curve (AUC).

*Size normalization*

For the baseline DCE images, the tumor crops had a median size of 48×48×16 pixels. Based on this we calculated the resizing factors of 3 dimensions and used the minimum one to resize the baseline crop, so the resized crop would not exceed 48, 48, and 16 pixels in height, width, and depth, respectively. The relative tumor shape was maintained across the patients. We then used the same resizing factor for the patient’s tumor crop at C4, which helps maintain the relative size of the tumor between scans. Finally, to accommodate possible large crops at C4 after resizing, the resized tumor crops were zero-padded to the same size of 80×80×24 for the semiquantitative maps, and to the same size of 80×80×12 for the DWI.


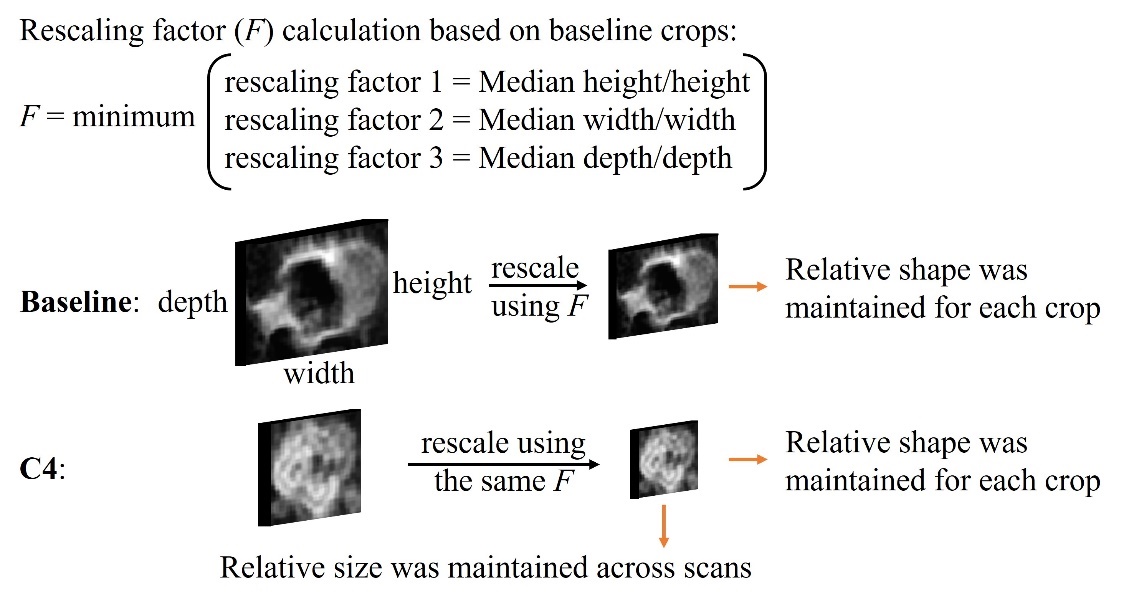


**SI Figure 1**. Illustration of the size rescaling process.

*Image combination for DL model development*

Besides using the combination of PEI map and b800 DWI for our DL model development, we investigated using different combinations of other images as the model inputs. The combinations tested were PEI only, b800 DWI only, MSI map with b800 DWI, and SER map with b800 DWI. The prediction AUCs of the training, validation, and retrospective independent testing groups are shown in the Supplementary Information (SI) Table 1.

**SI Table 1**. Area under the receiver operating characteristic curves (AUCs) for different image combinations used for deep learning model development.

|  | **Training AUC** | **Validation AUC** | **Testing AUC** |
| --- | --- | --- | --- |
| PEI only | 0.82 ± 0.24 | 0.72 ± 0.19 | 0.77 ± 0.17 |
| DWI only | 0.85 ± 0.20 | 0.74 ± 0.14 | 0.71 ± 0.16 |
| **PEI + DWI** | **0.97 ± 0.04** | **0.82 ± 0.10** | **0.86 ± 0.03** |
| MSI + DWI | 0.90 ± 0.17 | 0.71 ± 0.20 | 0.75 ± 0.14 |
| SER + DWI | 0.95 ± 0.04 | 0.70 ± 0.15 | 0.82 ± 0.05 |

**Note**: AUC: area under the curve. PEI: positive enhancement integral. DWI: diffusion weighted imaging. MSI: maximum slope of increase. SER: signal enhancement ratio.

The training, validation, and testing AUCs are consistently lower for the DL models trained with only one image set. The model trained with PEI and b800 DWI achieved the highest AUCs and the lowest standard deviations across the five folds for all the training, validation, and testing groups. The models trained with MSI or SER with DWI had numerically lower AUCs, especially in the validation group.

*DL model performance based on the standard definition of pCR*

The TNBC patients that had pCR in the breast but had residual cancer (residual burden level I or II) in axillary lymph nodes (ALNs) were excluded from our study because of the very small number (10 patients in total). Further, the pCR status of the primary tumor is predictive for the ALN status in breast cancer ^1^. However, being able to predict the treatment response for these patients is important because pCR in ALN was shown to be associated with improved overall survival in breast cancers ^2, 3^. Nevertheless, we retrained and re-evaluated the DL model by including the 10 patients who had breast pCR but residual ALN disease. 5 of these 10 patients that were enrolled early in the study were used for model development, and they were added to the five-fold cross-validation, with one patient per fold. Two patients were added to the retrospective independent testing group, and the remaining three who were enrolled later in the study (after the end of 2020) were included for the prospective blinded testing. Because using PEI map with b800 DWI achieved the best performance, we used the same image combination and the same network structure to retrain the DL model. The AUCs and other prediction metrics of the training, validation, retrospective independent testing, and prospective blinded testing groups are shown in SI Table 2.

**SI Table 2**. Deep learning model prediction results of the training, validation, retrospective independent testing, and prospective blinded testing groups based on the standard pCR definition.

| **Groups** | **AUC** | **Accuracy** | **Sensitivity** | **Specificity** | **PPV** | **NPV** |
| --- | --- | --- | --- | --- | --- | --- |
| Training | 0.96 ± 0.05 | 91 ± 6% | 92 ± 6% | 90 ± 6% | 90 ± 7% | 92 ± 5% |
| Validation | 0.78 ± 0.09 | 76 ± 6% | 83 ± 12% | 71 ± 12% | 74 ± 8% | 82 ± 13% |
| Testing (R) | 0.88 ± 0.02 | 81 ± 3% | 81 ± 9% | 82 ± 7% | 77 ± 5% | 87 ± 5% |
| Testing (P) | 0.76 ± 0.03 | 69 ± 3% | 60 ± 6% | 75 ± 7% | 65 ± 5% | 71 ± 2% |

**Note**: Testing (R): retrospective independent testing. Testing (P): prospective blinded testing. AUC: area under the curve. PPV: positive predictive value. NPV: negative predictive value.

After including these 10 patients, the model achieved an AUC of 0.96 in training and a similar prediction performance for the retrospective testing group compared to the breast pCR prediction (AUC 0.88 vs. 0.86). However, the model’s performance in the validation and the prospective testing groups became noticeably lower than those in the breast pCR prediction (AUCs of 0.78 vs. 0.82, 0.76 vs. 0.83, respectively). These findings suggest that it can be challenging for the current DL model to predict the disease status in ALNs using only the images of the primary tumors in the breast. It could also be a result of the very small number of the patients available with a breast pCR but residual ALN diseases. In the future, we plan to increase the number of these patients for model training and evaluation. Further, Sun et al. showed that the peritumoral images of ultrasound can be helpful to predict ALN metastasis for breast cancer ^4^. Similarly, Zheng et al showed that DL on conventional ultrasound images, shear wave elastography, and clinical information can predict breast cancer ALN metastasis ^5^. Therefore, to include the peritumoral MRI or ultrasound images for training may improve the performance of our current DL model.

**References**

1. Kuerer, H.M.*, et al.* Clinical course of breast cancer patients with complete pathologic primary tumor and axillary lymph node response to doxorubicin-based neoadjuvant chemotherapy. *J. Clin. Oncol.* **17**, 460-460 (1999).

2. Rouzier, R.*, et al.* Incidence and prognostic significance of complete axillary downstaging after primary chemotherapy in breast cancer patients with T1 to T3 tumors and cytologically proven axillary metastatic lymph nodes. *J. Clin. Oncol.* **20**, 1304-1310 (2002).

3. Mougalian, S.S.*, et al.* Ten-year outcomes of patients with breast cancer with cytologically confirmed axillary lymph node metastases and pathologic complete response after primary systemic chemotherapy. *JAMA Oncol.* **2**, 508-516 (2016).

4. Sun, Q.*, et al.* Deep learning vs. radiomics for predicting axillary lymph node metastasis of breast cancer using ultrasound images: fon't forget the peritumoral region. *Front. Oncol.* **10**, (2020).

5. Zheng, X.*, et al.* Deep learning radiomics can predict axillary lymph node status in early-stage breast cancer. *Nat. Commun.* **11**, 1236 (2020).
